# Relevant Sex Appeals in Advertising: Gender and Commitment Context Differences

**DOI:** 10.3389/fpsyg.2016.01456

**Published:** 2016-09-30

**Authors:** Even J. Lanseng

**Affiliations:** Department of Marketing, BI Norwegian Business SchoolOslo, Norway

**Keywords:** sex in advertising, gender differences, sociosexuality, commitment context, relevant sex appeals

## Abstract

This research investigates differences in men's and women's attitudes toward ads featuring product-relevant sex appeals. It is found that women, but not men, were more negative toward an ad featuring an attractive opposite-sex model when their commitment thoughts were heightened. Women were also more negative toward an ad with an attractive same-sex model in the presence of commitment thoughts, but only when they scored high on sociosexuality. Men appeared unaffected, regardless of their level of sociosexuality. Commitment thoughts were manipulated by two types of prime, a parenting prime (study1) and a romantic prime (study 2). Results are explained by differences in how men and women react to sexual material and by differences in men's and women's evolved mating preferences.

## Introduction

Both women's and men's attitudes toward ads containing irrelevant or overly explicit sex appeals (e.g., a straddling model promoting hamburgers) are typically negative because such content violates certain norms. Nevertheless, research has found that attitudes toward ads featuring irrelevant sex appeals are more positive in situations which facilitate intuitive instead of reasoned thinking (Sengupta and Dahl, 2008; Dahl et al., 2009). The reason is that detecting that a certain norm is violated by the ad content requires reasoned thinking (Campbell and Kirmani, 2000). Further, it was found that when reasoned thinking is restricted, men's attitudes toward irrelevant sex ads are more positive than women's (Sengupta and Dahl, 2008; Dahl et al., 2009), but that women's attitudes become more positive and approach men's attitudes when perceived within a relationship-commitment frame (Dahl et al., 2009). Also, among women with more positive attitudes toward sex *per se*, attitudes toward ads featuring irrelevant sex are similar to those of men (Sengupta and Dahl, 2008). Both evolutionary and social explanations propose that women appreciate sex when perceived within a frame of relationship commitment whereas men appreciate sex whether relational or casual (Trivers, 1972; Buss, 1998; Schwartz and Rutter, 1998; Baumeister and Twenge, 2002; Hill, 2002). Evolutionary and social accounts also suggest that men, on average, hold more positive attitudes toward sex itself than women do (Griffitt and Kaiser, 1978; Malamuth, 1996). Consequently, women with attitudes that are more positive toward sex *per se* can be expected to hold attitudes toward ads featuring irrelevant sex that are similar to those of men.

The current research extends previous research by examining the effect of relationship commitment on ad attitudes when the sex appeal is relevantly connected to the advertised product and occurs in a situation that facilitates reasoned thinking. The current research also extends previous research by examining the joint effect on ad attitudes of relationship commitment and general desire toward sex.

In the research presented here, irrelevant and overly explicit sex appeals are referred to as gratuitous sex appeals (Sengupta and Dahl, 2008) and relationship commitment is defined as a commitment between two persons in a long-term relationship in which both parties invest resources, such as parenting and romance (Dahl et al., 2009).

In situations with relevant or non-gratuitous sex appeals and reasoned thinking, thoughts about committed relationships are likely to lead to less favorable ad attitudes among women. Generally, sex that is relevant for the advertised product is likely to be accepted because sex is then seen as part of the product argument. When thoughts about relationship commitment are evoked and reasoning is possible, women are likely to have a different perspective than men on sex appeals. Reasoned thinking is likely to allow for women's tendency to inhibit themselves in the presence of sexual content (Geer and Bellard, 1996; Geer and Melton, 1997) and then to be attuned to cues of a man's nurturing abilities because these are qualities that women search for rationally when they contemplate relationship commitment. When these cues are not readily available in the ad, women's response to sexual ad content is negative and this response is likely to spill over into negative ad attitudes. Relationship commitment thoughts are not expected to change men's perspectives. Like women, men should also accept sexual content when it is a relevant product argument in an ad. Commitment thoughts, however, do not change this acceptance. Unlike women, men are attuned to sexual attractiveness when thinking about relationship commitment.

The current research also proposes that women with high sexual desire hold attitudes toward gratuitous sex appeals than are more negative than do women with low sexual desire when relationship commitment thoughts are heightened. This difference is because high-desire women are more sensitive to competition from other women. Men's attitudes toward sex-appeal ads will remain unaffected by heightened commitment thoughts and levels of sexual desire. This lack of difference is because men tend to be competitive irrespectively of commitment thoughts and because they vary less than women in levels of sexual desire.

Two experimental studies are presented in which relationship commitment is primed by evoking thoughts about parenting and romantic arousal, two important ingredients in relationship commitment. The experimental results generally support the reasoning above.

Results should be relevant to advertisers and policy makers that want to convey persuasive messages. Also when a sex appeal is relevant to a message, there are limits to people's acceptance of the appeal.

## Conceptual idea

In two papers Dahl and colleagues (Sengupta and Dahl, 2008; Dahl et al., 2009) have addressed men's and women's reactions to gratuitous sex appeals in advertising. They found that men are more positive than women are toward such appeals, but that evoked relationship commitment thoughts and positive attitudes toward sex *per se*, make women become more positive toward gratuitous sex appeals.

Dahl et al. (2009) showed that women's attitudes toward gratuitous sex in an ad were more favorable if the ad was shown within a commitment-laden frame. In one experiment in which the processing of ad information was restricted (high cognitive load) women's attitudes toward an ad depicting a wristwatch accompanied by a nude and copulating couple were more favorable when the watch was framed as a gift from a man to a woman (commitment cue) than when it was not. Men's attitudes were more positive when the watch was not positioned as a gift. The results for women were corroborated in another, all-female-participants experiment in which relationship commitment was manipulated by means of priming participants with a committed vs. an uncommitted romantic partner.

Sengupta and Dahl (2008) demonstrated that in circumstances in which the processing of ad information was restricted (high cognitive load), attitudes toward an ad featuring gratuitous sex appeal (wristwatch accompanied by a nude and copulating couple) and attitudes toward a product-relevant sex appeal (condoms accompanied by a nude and copulating couple) were the same. This pattern was the same for men and women, but men had attitudes that were more positive than those of women for both ads.

Sengupta and Dahl (2008) also found that women's general attitudes toward sex moderated their reactions toward gratuitous sex in ads. In particular, women with less positive attitudes toward sex held a more favorable attitude toward the nonsexual ad (a wristwatch accompanied by mountain scene) than toward the sexual ad (a wristwatch accompanied by a nude and copulating couple). Conversely, women with attitudes that were more positive toward sex held attitudes that were more positive toward the sexual ad than toward the nonsexual ad. In fact, these women held attitudes toward gratuitous sex appeals that are similar to those of men.

The explanation for the results reviewed above starts with constrained processing, which leads to spontaneous reaction. The *relevance* of the sexual content therefore plays a minor role and is less likely to violate norms (Dahl et al., 2009). In the absence of norms, gender differences with respect to attitudes toward sexual content are manifested. Men tend to have a more recreational orientation toward sex and a more positive attitude toward sex *per se*. Women tend to have a more commitment-oriented approach to sex and generally hold more negative attitudes toward sex *per se* (Hill, 2002). Evolutionary psychology explains these gender differences by differential parental investment. Women have to invest greater resources in bringing a child to birth and in nurturing it than men do. Women are therefore more selective than men are in their mating considerations and look for partners who are likely to commit resources to carry their part of the burden (Trivers, 1972; Buss, 1998). Because men have evolved a recreational orientation toward sex, they are also inclined to hold positive attitudes toward sex and portrayals of sex in general (Malamuth, 1996). Because women have evolved an orientation toward committed relationship and away from casual sex, they are likely to hold attitudes that are more negative toward sex and sexual portrayals than men are.

Socialization-based explanations suggest that social influence from peers, government, religion, and other sources is heavily biased toward promoting a recreational orientation toward sex for men and a relationship orientation for women (Schwartz and Rutter, 1998; Baumeister and Twenge, 2002). Further, the socialization process typically exposes girls and boys to different messages and differential message valence about the same sexual stimuli. This social conditioning, in turn, leads girls and boys to develop different attitudes toward sex and sexual stimuli (Griffitt and Kaiser, 1978). Although men on average hold more positive attitudes toward sex than women do, some women are more positive than others are. Such intragender variation may stem from differences in women's childhood experiences, material resources, physical attractiveness, etc. (Sengupta and Dahl, 2008). Intragender variation also exists for men, but empirical evidence suggests that this variation is more pronounced for women (Lippa, 2009).

The findings reviewed above occurred in a situation where participants' cognitive processing was constrained. Under this condition, the relevance of the sexual content or the lack of it is unlikely to play a part in the judgment (Dahl et al., 2009) and attitudes toward the ad are likely to be relatively favorable. Because irrelevancy is simply less of an issue when the sexual content and the advertised product are connected, attitudes toward ads with such connected sexual content should be relatively positive even if processing is unconstrained.

What about when processing is unconstrained and the sexual content is perceived to be connected to the advertised product? Are ads always effective when the sexual content appears in relation to products like sun lotions, underwear, or hygiene items, or are attitudes different between men and women and across commitment conditions?

Men and women are likely to differ in their attitudes toward ads featuring relevant sexual content when their processing is unrestricted. Because sexual behavior, evolutionarily speaking, implies important concerns (e.g., reproduction, parental investment), attentional and conscious cognitive mechanisms, including inhibitory mechanisms, are likely to be triggered by sexual images (Gross, 1998; Spiering et al., 2004). The activation of conscious mechanisms should be facilitated in the absence of factors demanding cognitive and attentional resources, such as experimenter-imposed cognitive load. An example of a conscious inhibitory mechanism in the context of sexual ad content is the phenomenon known as sexual content–induced delay (SCID) studied by Geer and colleagues (Geer and Bellard, 1996; Geer and Melton, 1997). SCID refers to hesitancy in people's decision-making when they are exposed to sexual material. People are less quick to recognize and process sexual information than nonsexual information.

SCID is found to be more pronounced for women than for men. In lexical decision tasks that included sexual, romantic, and neutral words, women, but not men, displayed longer decision times when the target words were sexual. A similar, but smaller effect was also found for romantic words (Geer and Manguno-Mire, 1996). Rupp and Wallen (2008) reviewed findings that are consistent with these gender differences in SCID. Among reported gender differences were different gaze patterns, with men more focused on the sexual stimulus itself (e.g., body parts) and women paying more attention to contextual information (e.g., clothing), and women are less likely to become habituated to stimuli, thus remaining attentive and interested for longer than men do.

A gender difference in SCID makes evolutionary sense. Unlike men, who maximize their mating opportunities, women, who invest more in offspring care and bear the burden during pregnancy, have more to lose from mating indiscriminately (Bailey et al., 1994). Therefore, when commitment thoughts are heightened in the presence of sexual content, women will regulate their response. Particularly, it is expected that a woman will search for information cues that are diagnostic of whether a man can share her burden, such as his popularity, wealth, or social aptness. Although to a lesser extent than women do, men will also regulate their response to sexual content. Men's particular search, however, is focused on women's sexual attractiveness.

The above predictions suggest that women and men on average have differential attitudes toward sexual ad content. Nevertheless, neither evolutionary nor social determinism is likely to represent the whole truth about these differences. Not all people are nudged in the same direction by relationship commitment thoughts when judging sexual content. Individual differences are likely to play a role. Research that dates back to Alferd Kinsey in the nineteenth century (Kinsey et al., 1948, 1953) suggests that people differ with respect to how sexually restricted they are. As conceptualized by Simpson and Gangestad (1991), sociosexuality refers to individual variation in the willingness to engage in sexual activities outside committed relationships. The same authors developed the Sociosexual Orientation Inventory (SOI) scale to assess sociosexuality along a restricted–unrestricted continuum. Restricted people score low on the SOI scale and are inclined to engage in sexual activities exclusively in emotionally close and committed long-term relationships. Unrestricted people score high on the SOI scale and show a tendency to engage in sexual activities with low commitment and investment, and have a short-term perspective about changing partners. The most recent development of the sociosexuality concept suggests that it consists of three sub-dimensions, attitude, desire, and behavior (Penke and Asendorpf, 2008). Sex differences are more pronounced for desire than for attitude and behavior, and men are generally more unrestricted than women are on the SOI scale (Schmitt, 2005, 2007).

Although, evolutionary psychology underlies the predictions made in the current research, these predictions are also consistent with socialization-based explanations for women's and men's reactions to sexual stimuli. Differences in society's role expectations and in social influence, from parents, peers, and other significant influencers, are biased toward promoting male sexuality and undermining female sexuality (Schwartz and Rutter, 1998; Baumeister and Twenge, 2002), and are likely to shape women's more negative and men's more positive reactions and approaches to sexual stimuli (Griffitt and Kaiser, 1978).

## Predictions

Using the literature reviewed above, this research suggests that for advertising involving relevant sex appeals and unrestricted cognitive processing, commitment and sociosexuality will interact to create results that diverge from those reported for advertising involving gratuitous sex appeals and restricted cognitive processing. Because it is reasonable to believe that men's and women's ad attitudes will depend on whether the sex appeal involves male or female nudity, hypotheses are derived for both male and female nudity.

### Opposite sex

When the sexual content is relevant for the advertised product, consumers should be mildly positive toward it because it acts as a strong argument (see Petty et al., 1981 for a discussion of strong arguments). Nevertheless, given that consumers are cognitively unrestricted, the concept SCID suggests that they are likely to regulate their response to sexual material. In the context of relationship commitment, men and women regulate differently. Women are oriented toward offspring care and will value cues to that. If they instead encounter male nudity in an ad, they will experience a mismatch between stimuli and their current commitment thoughts, which leads to negative ad attitudes. Men are oriented toward physical characteristics irrespectively of commitment thoughts, and will generally value female nudity positively. Therefore,

**H1:** Women's attitudes toward ads featuring male nudity are less positive when their commitment thoughts are heightened than when not. Men's attitudes toward ads featuring female nudity will remain unaffected by heightened commitment thoughts.

### Same sex

Because women invest more in parenting than men do, they have more to lose from bad mating choices, and they have therefore become the choosier sex (Trivers, 1972; Buss, 1996). Because women can be more selective, men have become the more competitive sex (Buss and Schmitt, 1993). Although, commitment thoughts may evoke competitiveness in women, women are the choosier sex, so commitment thoughts may not automatically affect their competitiveness. Women with high scores on sociosexuality are more sensitive to intrasex competition, because competition from other women is more salient to them. Hence, these women are likely to be more competitive in a commitment context including being more negative toward other women's physical attractiveness.

Men are generally more competitive than women are. Hence, men must engage in more intrasex competition than women do. Since men tend to have a recreational orientation toward sex, commitment is unlikely to influence their view on competition. Men are competitive with respect to both short-term and long-term relationships. So, regardless of commitment thoughts, men will dislike attractiveness in other males. Further, men vary less in levels of sociaosexuality than women do (Lippa, 2009). Therefore,

**H2:** Women with high levels of sociosexuality will have less positive attitudes toward ads featuring female nudity when their commitment thoughts are heightened, whereas women with low levels of sociosexuality will have attitudes that are more positive. Men's attitudes toward ads featuring male nudity will remain unaffected by heightened commitment thoughts and levels of sociosexuality.

## Study 1

To test the predictions that women exhibit more positive attitudes toward ads with product-relevant sexual content under normal circumstances than in conditions with heightened accessibility of relationship-commitment thoughts, and to test the moderating role of sociosexuality, an experimental study was conducted.

### Participants

Amazon's crowdsourcing platform Mechanical Turk (MTurk) was used to collect data for the experiment. Although the typical MTurk sample is not demographically representative of the American population, it provides substantial variation along important demographic variables such as age, gender, income, education, employment situation, ethnicity, and religious beliefs (Huff and Tingley, 2015; Levay et al., 2016). This variation was deemed necessary because an important variable in the present research is sociosexuality, a variable that develops differently in the population on several of these demographic variables. Generally, a MTurk sample can be expected to contain slightly more educated people who have a slightly lower income than people in a sample from the population-based American National Election Studies (Levay et al., 2016). Further, a MTurk sample has a number of strengths compared to a laboratory convenience sample, including heterogeneity, low coverage error, and few experimenter effects (Paolacci et al., 2010).

The sample consisted of 170 MTurk participants (85 female). Participants were between 18 and 66 years old with an average of 34.3 years (*sd* = 11.5), which is close to what could be expected from a MTurk sample (*m* = 31.6). Participants had an approval rate of at least 95% (i.e., 95% or more of that participant's previous submissions were approved by requesters), which is the default MTurk cutoff. Participants received $1 for completing the study.

### Stimuli

Stimuli were two fictitious ads for the sunscreen brand Dr. Martins of Maui (Appendix). One ad featured an attractive male model wearing swimming shorts and the other ad displayed an attractive bikini-clad female model. Ads were designed to portray an attractive male and an attractive female model, but in a product-relevant (non-gratuitous) context. Models wearing a bikini or trunks are relevant for a sun lotion brand. To further facilitate the testing of the hypotheses, physical attractiveness was salient in both models whereas cues to popularity, wealth, social aptness, etc. were absent. Except for the models, all ad elements were the same for both ads. Both models were previously tested and found to be equally attractive (Majoor, 2011).

### Design and procedure

To evoke relationship-commitment thoughts, a priming procedure was used. Priming refers to the process whereby exposure to a certain experience subsequently increases the accessibility of a conceptual category, thereby increasing the likelihood of that category being used to encode and respond to new information (Fiske and Taylor, 1991). Priming experiments typically expose participants to a stimulus called prime (the particular experience) that evokes associations in memory (a particular conceptual category) followed by exposure to a seemingly unrelated target stimulus. In this experiment, participants were exposed to *parenting* by thinking and writing about benefits of having children. After this exposure (prime), the concept of relationship commitment (conceptual category) is likely to be accessible and salient in participants' memory and, in turn, is readily available to be used by participants to judge the ads and their content (target).

A 2 (Gender: men vs. women) × 2 (Commitment prime: parenting vs. happiness) between-subjects design was employed. The first factor was participants' recorded sex. The second factor was priming of relationship commitment. Participants were randomly assigned to either a parenting priming condition or a happiness control condition. These priming tasks were adapted from tasks used in previous studies (e.g., Maner et al., 2007).

Participants in the parenting prime responded to the following instructions: “Think about the benefits of having children and how that will make you feel good. Write down four or five such benefits.” “Now, try to visualize the greatest of these benefits that you just wrote down and describe in detail how this benefit makes you feel good.”

Participants in the control condition responded to the instructions: “Think about being happy. Write down four or five times you have been very happy in life.” “Now, try to visualize the time that you were the most happy, and describe in detail about this time.” Happiness was chosen over an “empty” or dull control task to minimize the possibility that differences in ad attitudes between the treatment and control conditions could be explained by arousal or emotional differences in participants. The descriptions provided by the participant in the priming tasks were in place to strengthen the manipulation and these descriptions were not subject to any further analysis.

Immediately after the priming task, participants completed the 16-item Brief Mood Introspection Scale (BMIS; Mayer and Gaschke, 1988). This measure was used in a mediation analysis to assess whether obtained results were driven by affect.

After the BMIS items, participants saw the ads and evaluated them on four items (liking, appeal, attractiveness, pleasantness). All items were measured on seven-point bipolar scales (e.g., like/dislike). In the subsequent analysis, these items were combined into two attitude indexes, attitude toward a male model ad (α = 0.95) and toward a female model ad (α = 0.94), and served as dependent variables.

Finally, sociosexuality was measured using the 9-item revised Sociosexual Orientation Inventory, SOI-R (Penke and Asendorpf, 2008). This scale has three sub-dimensions: behavior (number of partners last 12 months, number of one-night-stand partners, number of partners without having long-term interests), attitude (sex without love is ok, comfortable with causal sex, certainty about long-term relations), and desire (fantasies about uncommitted sex, sexual arousal evoked by non-partners, fantasies about spontaneous sex with strangers). Given previous research on this scale (Penke and Asendorpf, 2008), it was expected that the differences between men and women would be more pronounced with respect to the desire dimension.

### Results

As one of the hypotheses involves the metrically scaled moderator variable sociosexuality, a regression-based approach (Process, Hayes, 2013) that avoids dichotomization, was chosen to test hypotheses. Regression analyses were run for each of the dependent variables—attitude toward the ad featuring a male model and attitude toward the ad featuring a female model. To test H1, the attitude variables were regressed on the two independent dichotomous variables, parenting thoughts and the viewer's sex. To test H2, a third independent metrically scaled variable—sociosexuality—was included in the regression equation. In all regressions, viewers' age was a covariate. For simplicity, figures containing only non-significant results will not be displayed.

#### Opposite sex

**Figure 1:**
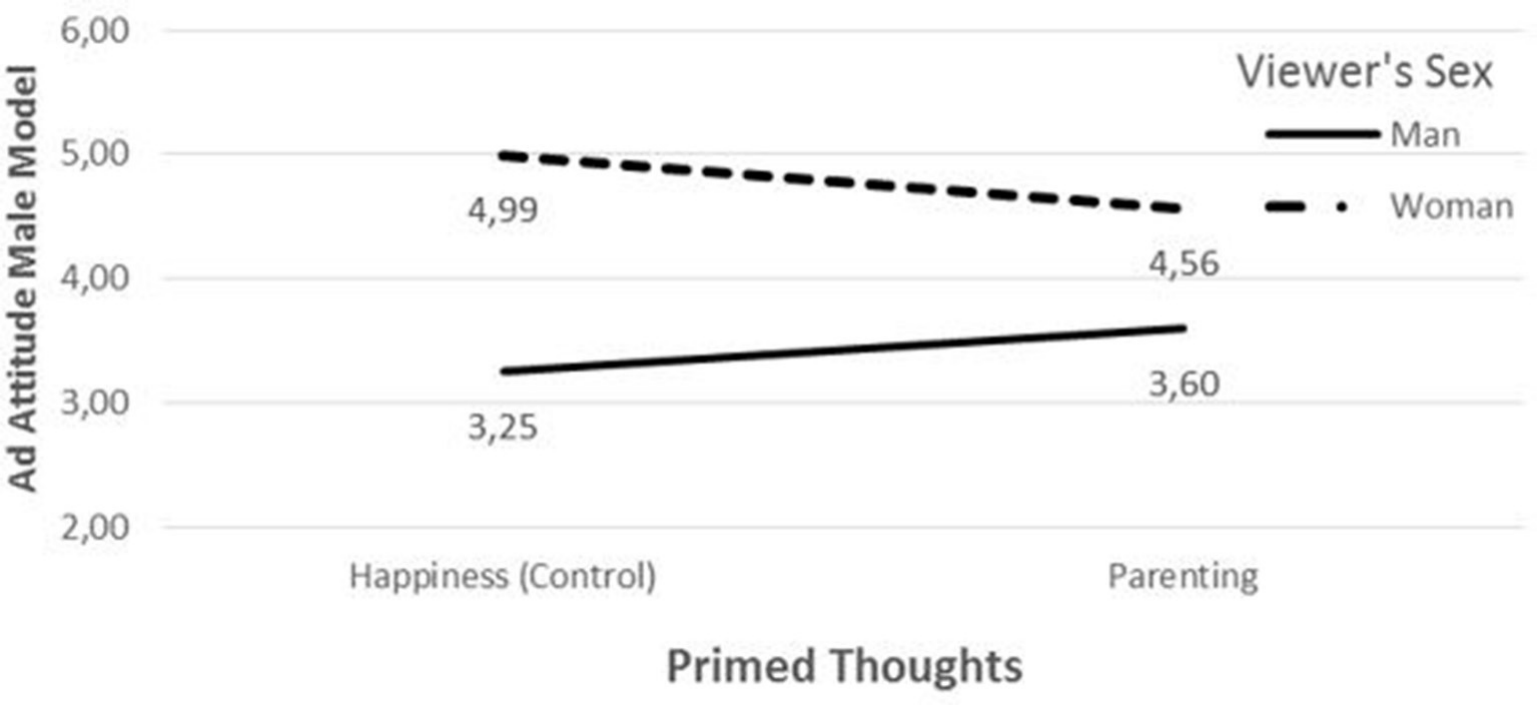
**Attitude toward ad featuring male model by parenting thoughts and viewer's sex**.

A statistically significant interaction effect between parenting and viewer's sex on attitude toward the male model ad was observed [*t* = 1.84, *p* = 0.03 (one-tailed)]. Results are shown graphically in Figure 1. Attitude toward the ad featuring the male model is measured on the Y-axis and relationship commitment primed by parenting vs. happiness (control) thoughts appears on the X-axis. The solid line shows attitude level for men (male experimental participants) and the dashed line shows attitude level for women (female experimental participants). If the solid and dashed lines have different patterns across the two different thought conditions, they indicate an interaction effect (parenting thoughts have differential effects on men's and women's ad attitudes).

As can be seen from Figure 1, women's attitude toward the male model is more negative in the parenting than in the happiness condition, although only marginally so [Ŷ_parenting_ = 4.56 vs. Ŷ_happiness_ = 4.99, *t* = −1.42, *p* = 0.08 (one-tailed)]. This observation lends marginal support to the first part of H1. Men's attitude was equal in both conditions [Ŷ_parenting_ = 3.60 vs. Ŷ_happiness_ = 3.25, *t* = 1.19, *p* = 0.24 (two-tailed); Figure 1]. The interaction between commitment and viewer's sex on attitude toward the ad featuring the female model was insignificant [*t* = 0.26, *p* = 0.80 (two-tailed)]. In particular, men's attitude toward this ad did not differ across the conditions [Ŷ_parenting_ = 5.18 vs. Ŷ_happiness_ = 5.14, *t* = 0.14, *p* = 0.89 (two-tailed)]. This observation supports the second part of H1.

These results lend weak support to H1, stating that women's attitudes toward ads with male nudity are less positive when their commitment thoughts are heightened, and that men's attitudes toward ads featuring female nudity remain unaffected by such thoughts.

#### Same sex

**Figure 2:**
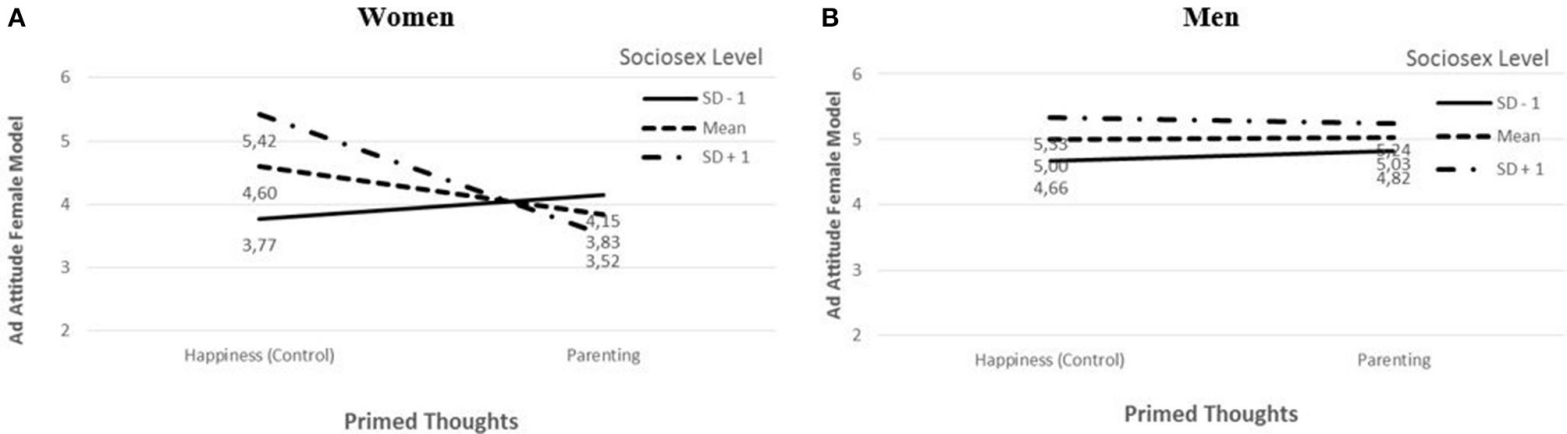
**Attitude toward ad featuring female model by parenting thoughts and level of sociosexuality for women (A) and men (B)**.

A statistically significant three-way interaction between parenting, sociosexuality (sub-dimension desire), and viewer's sex was observed for attitudes toward the female model ad [*t* = 1.70, *p* = 0.05 (one-tailed)]. A “spotlight” analysis (see, e.g., Fitzsimons, 2008) of this interaction shows that parenting thoughts and sociosexuality had an interactive effect on ad attitudes for women [*t* = −2.29, *p* = 0.01 (one tailed)], but not for men [*t* = −0.35, *p* = 0.36 (one tailed)]. For the lower level of sociosexuality (1 standard deviation below the mean), women's ad attitudes did not differ between the parenting and the control conditions (SD −1: Ŷ_parenting_ = 4.15, Ŷ_happiness_ = 3.77, *t* = 0.99, *p* = 0.16; Figure 2). For the medium level of sociosexuality (mean), attitude was lower in the parenting than in the control condition (Mean: Ŷ_parenting_ = 3.83, Ŷ_happiness_ = 4.60, *t* = −1.79, *p* = 0.04; Figure 2). For the higher level (1 standard deviation above mean), attitude was also lower in the parenting than in the control condition (SD +1: Ŷ_parenting_ = 3.52, Ŷ_happiness_ = 5.42, *t* = −2.25, *p* = 0.01; Figure 2). These observations support the first part of H2.

No significant three-way interaction was observed for attitude toward the male model [*t* = 0.85, *p* = 0.20 (one tailed)]. In particular, men's attitudes toward this same-sex ad did not differ significantly across the conditions at any of the sociosexuality levels [SD −1: Ŷ_parenting_ = 3.36, Ŷ_happiness_ = 3.16, *t* = 0.32, *p* = 0.75 (two-tailed)], [Mean: Ŷ_parenting_ = 3.50, Ŷ_happiness_ = 3.22, *t* = 0.76, *p* = 0.45 (two tailed)], or [SD +1: Ŷ_parenting_ = 3.64, Ŷ_happiness_ = 3.28, *t* = 1.08, *p* = 0.28 (two tailed)]. These observations support the second part of H2.

In summary, results support H2. When their commitment thoughts are heightened, women with higher levels of sociosexuality have less positive attitudes toward ads featuring same-sex nudity than women with low levels do. Men's attitudes toward same-sex nudity ads are unaffected by heightened commitment thoughts and levels of sociosexuality.

To control that the observed effect on attitude for the male model ad is not working through affect, a mediation analysis was performed. Sengupta and Dahl (2008) performed a similar analysis to confirm that affect was a mediator. In this analysis, we expect affect to not be a significant mediator, because conscious regulatory mechanics are operating instead. A regression equation model (Process, Hayes, 2013) in which positive and negative moods were specified to mediate the effect of the commitment prime on attitudes toward the female model, yielded non-significant results. The parenting prime affected neither positive [*b* = −0.01, *p* = 0.46 (one-tailed)] nor negative [*b* = −0.09, *p* = 0.14 (one-tailed)] moods. Nor did parenting influence attitude when positive and negative moods were added to the equation [*b* = −0.22, *p* = 0.18 (one-tailed)].

Affect also played no mediating role in producing the results for attitudes toward the male model ad. As reported above, the prime affected neither positive [*b* = −0.01, *p* = 0.46 (one-tailed)] nor negative [*b* = −0.09, *p* = 0.14 (one-tailed)] moods. Nor did the prime affect attitude when positive and negative moods were added to the equation [*b* = −0.08, *p* = 0.35 (one-tailed)].

These results are consistent with the assumptions that in the absence of factors placing demand on cognitive resources, affect will not drive the results. Hence, it is more likely that deliberate mechanisms have been involved in producing the results.

### Discussion

Results gave support to both hypotheses. For ads in which the sex appeal is related to the product, attitudes are less positive for women when they are primed with parenting thoughts. When no particular restrictions are placed on participants' cognition and sexual content is present, SCID is present. SCID differs for women and men. Consistent with predictions of evolutionary psychology, men find what they want in an ad with sexual opposite-sex content and carry on to form positive attitudes, whereas women regulate their judgments differently and will find a mismatch between their current commitment thoughts and the sexual opposite-sex ad content. This mismatch is likely to produce negative attitudes.

Findings are consistent with those of previous research that has shown that priming parenting led women to adopt a mate preference for socially dominant men, but that the parenting prime had no effect on men's mate preference for physically attractive women. The likely explanation for this gender difference is that parenting has little effect on men's preference for physical attractiveness because this preference is always strong. Men's reproductive fitness is constrained by female fertility whether they would like to commit long-term or have a more short-term focus (Buss and Schmitt, 1993). A woman's fertility influences a man's ability to produce viable offspring in both cases (Millar and Ostlund, 2006).

Furthermore, results suggest that sociosexuality plays a moderating role in women's attitudes toward same-sex sexual content when parenting thoughts are heightened. Women who are more open to short-term mating are also more sensitive to competition from other women. Attractive female models remind them about competition, which spills over to negative attitudes toward ads featuring these models.

## Study 2

Because this research suggests that commitment contributes to ad attitudes that deviate from what is found in previous research, it is important to investigate if results are specific to the commitment prime used in study 1. To check that the results are robust, study 2 investigates whether results also appear with a different commitment prime. Romantic arousal was used because it primes a slightly different facet of relationship commitment than parenting does.

### Design and procedure

A 2 (Gender: men vs. women) × 2 (Commitment prime: romantic arousal vs. happiness) between-subjects design was employed. In the romantic arousal condition, participants responded to the following instructions: “Think about romance. Write down four or five instances in which you have been romantically aroused.” “Now, try to visualize the instance in which you were the most romantically aroused, and describe in detail about this instance.” The control condition was identical to that of study 1. The procedure was identical to that of study 1.

### Participants and stimuli

The sample consisted of 85 MTurk participants (44 females). Participants were between 19 and 65 years old with an average of 33.2 years (*sd* = 10.3). Participants had an approval rate of at least 95%. Participants received $1 for completing the study. Participants in the happiness condition were a random sample of half of the control group participants from study 1. A sample, rather than the whole group, was used to make the romantic arousal and the happiness conditions more equal in size. The control group and the treatment group were both drawn from the same crowdsourcing platform (MTurk) simultaneously, so it is fair to say that they are both drawn from the same MTurk population.

Ads were the same as those used in study 1.

### Results

#### Opposite sex

**Figure 3:**
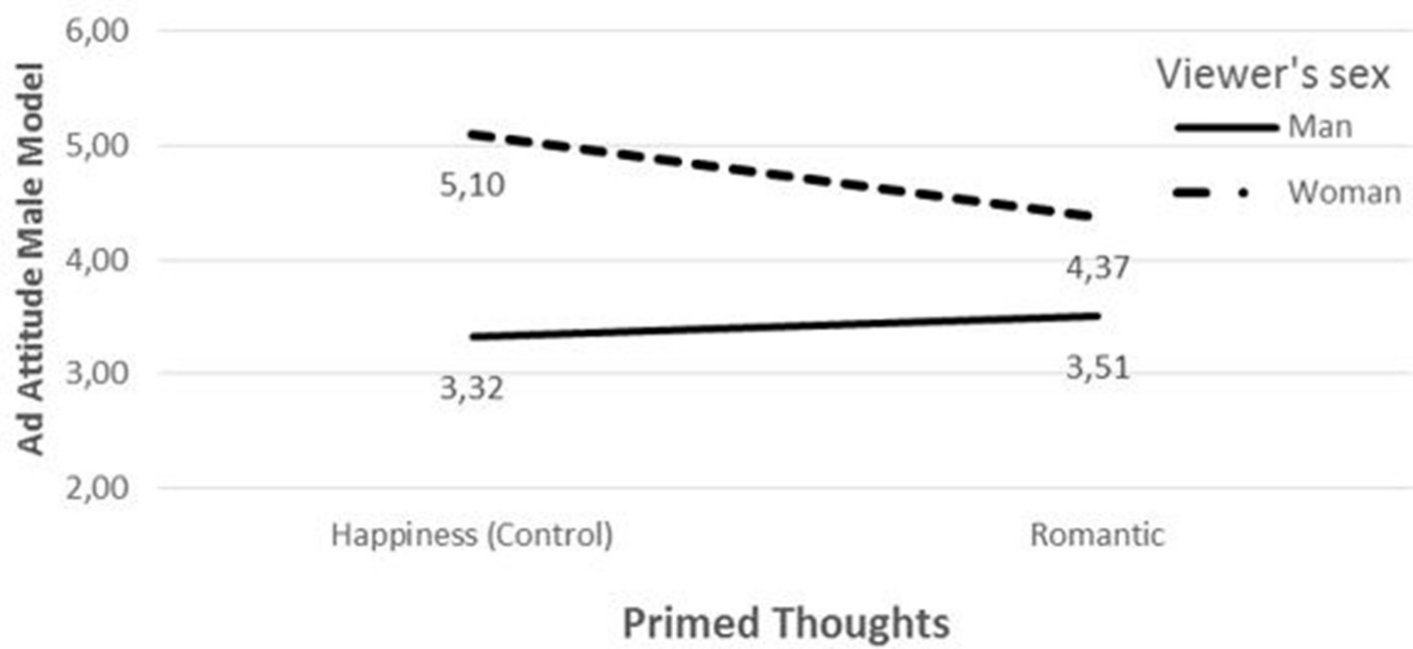
**Attitude toward ad featuring male model by romantic thoughts and viewer's sex**.

A marginally statistically significant interaction effect between the romantic prime and viewer's sex on attitude toward the male model ad was observed [*t* = 1.65, *p* = 0.05 (one-tailed)]. Women's attitudes toward the male model ad were more negative in the parenting condition than in the happiness condition [Ŷ_romantic_ = 4.37 vs. Ŷ_happiness_ = 5.10, *t* = −1.86, *p* = 0.03 (one-tailed); Figure 3]. This observation lends marginal support to H1's first part. Men's attitudes were equal in both conditions [Ŷ_romantic_ = 3.51 vs. Ŷ_happiness_ = 3.32, *t* = 0.48, *p* = 0.63 (two-tailed); Figure 3]. The interaction between commitment and viewer's sex on attitude toward the female model ad was insignificant [*t* = 0.42, *p* = 0.68 (two-tailed)]. Particularly, men's attitudes toward this ad did not differ across the conditions [Ŷ_romantic_ = 5.40 vs. Ŷ_happiness_ = 5.48, *t* = −0.17, *p* = 0.87 (two-tailed)], an observation that supports the second part of H1.

These results lend support to H1 and corroborate results from study 1 by means of an alternative priming of commitment thoughts.

#### Same sex

**Figure 4:**
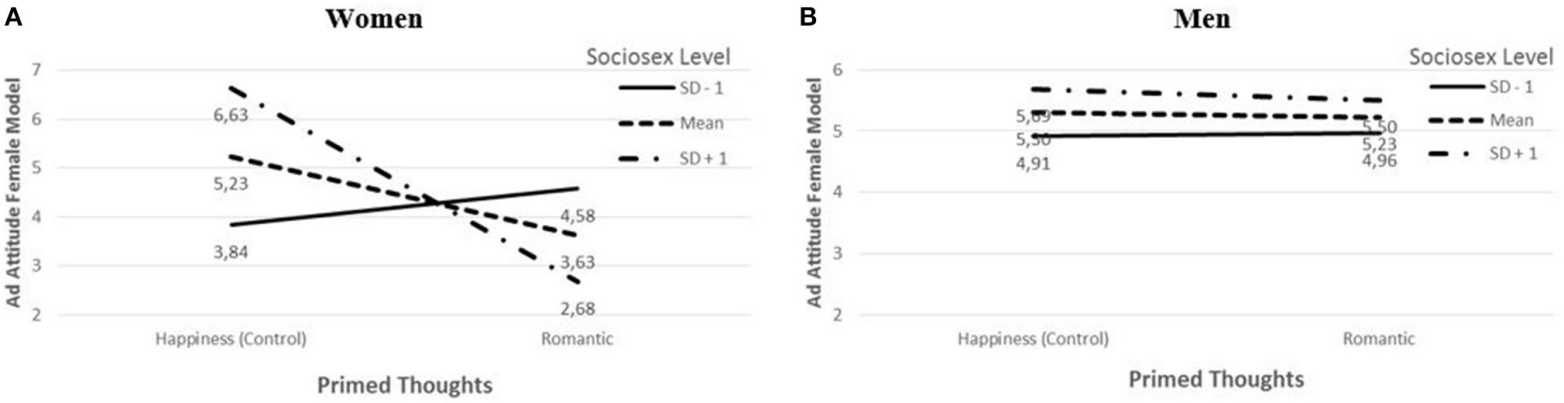
**Attitude toward ad featuring female model by romantic thoughts and level of sociosexuality for women (A) and men (B)**.

A statistically significant three-way interaction between the commitment prime, sociosexuality (sub-dimension desire), and viewer's sex was observed for the female model ad [*t* = 2.51, *p* = 0.01 (one-tailed)]. A “spotlight” analysis of this interaction shows that romantic thoughts and sociosexuality had an interactive effect on ad liking for women [*t* = −2.99, *p* = 0.00 (one-tailed)], but not for men [*t* = −0.29, *p* = 0.39 (one-tailed)]. For the lower level of sociosexuality (1 standard deviation below the mean), ad attitude was marginally higher in the romantic condition than in the happiness (control) condition (SD −1: Ŷ_romantic_ = 4.58, Ŷ_happiness_ = 3.84, *t* = 1.34, *p* = 0.09; Figure 4). For the medium (Mean: Ŷ_romantic_ = 3.63, Ŷ_happiness_ = 5.23, *t* = −2.61, *p* = 0.01; Figure 4) and for the higher level of sociosexuality (SD +1: Ŷ_romantic_ = 2.68, Ŷ_happiness_ = 6.63, *t* = −3.04, *p* = 0.00; Figure 4), attitude was higher in the control condition than in the romantic condition. These observations support the first part of H2.

The three-way interaction for the male model ad was not statistically significant [*t* = 0.40, *p* = 0.34 (one-tailed)]. Particularly, men's attitudes toward the male ad did not differ significantly across the conditions at any of the sociosexuality levels [SD −1: Ŷ_romantic_ = 3.81, Ŷ_happiness_ = 2.80, *t* = 1.38, *p* = 0.17 (two-tailed)], [Mean: Ŷ_romantic_ = 3.63, Ŷ_happiness_ = 3.16, *t* = 1.04, *p* = 0.30 (two-tailed)], or [SD +1: Ŷ_romantic_ = 3.46, Ŷ_happiness_ = 3.51, *t* = −0.13, *p* = 0.90 (two-tailed)]. These observations support the second part of H2.

Taken together, results both support H2 and corroborate results from study 1 by means of another commitment thoughts prime.

Again, an analysis was performed in which positive and negative moods were specified to mediate the effect of the commitment prime on attitude toward the female model ad. The romantic prime affected neither positive [*b* = 0.02, *p* = 0.45 (one-tailed)] nor negative [*b* = 0.04, *p* = 0.38 (one-tailed)] moods. Nor did the prime influence attitude when positive and negative moods were added to the equation [*b* = −0.21, *p* = 0.26 (one-tailed)]. Similar non-significant results were obtained for the male model ad. Hence, it is concluded that the effect of heightened romantic thoughts on attitude toward the female model ad is not working through affect.

### Discussion

Results corroborate those of study 1. In addition, these results underscore the role played by evolutionary psychology in peoples' ad attitudes. Men and women hold attitudes that are consistent with their evolved mating preferences. Men's attitudes appear to be unaffected by a parenting prime. Parenting is less of an investment for men so they can afford to value sexual attractiveness, just as much as they do in the non-parenting situation. Women's attitudes are different. Parenting implies huge investments in time, money, and offspring care; hence, women cannot afford to place too much value on sexual attractiveness. They therefore evaluate sexual attraction in ads less favorably in parenting situations than in non-parenting situations. Men's ad attitudes appear to be unaffected also by romantic thoughts. Sexual attractiveness is what men pursue when thinking about mating and they pay little attention to the costs of pregnancy. Hence, they can afford to value sexual attractiveness in romantic as well as non-romantic situations. Women cannot look away from the costs of pregnancy in romantic situations. They will therefore consider cues to financial strength and social aptness of men, not only sexual attractiveness. Sexual attractiveness is easier for women to value in non-romantic situations. These findings are consistent with those of previous research suggesting that men's preferences for attractive women are hardly affected by contextual factors (Kenrick et al., 1993) but that women's preferences are rather flexible in the face of changing contexts (Baumeister, 2000).

## General discussion

Research has shown that when processing is restricted, men and particularly women are negative toward gratuitous sex appeals in advertising, but that attitudes that are more positive emerge with attitudes that are more positive toward sex *per se* or when commitment thoughts are heightened (Sengupta and Dahl, 2008; Dahl et al., 2009). It might therefore be tempting to conclude that a non-gratuitous (brand-relevant) sex appeal will contribute to ad attitudes that are even more positive. After all, the so-called match-up hypothesis predicts that ads with sexual appeals that are relevant for (match) the advertised brand are evaluated more favorably than ads with irrelevant sexual content are (Solomon et al., 1992; Till and Busler, 2000). Results from this research, however, show that the effect of brand-relevant sexual content on ad attitudes is less straightforward.

It is found that for ads in which the sex appeal is related to the advertised brand, attitudes are less positive for women when they are influenced by a commitment context. When cognitive load is low and sexual content is present, SCID is prevalent, and the results of SCID differ between women and men. In the context of heightened commitment thoughts, men find what they want in an ad with sexual content and carry on to evaluate it positively, whereas women will find a mismatch between their commitment thoughts and the sexual content in the ad. This mismatch produces negative attitudes toward *opposite-sex* models for women. A likely explanation for SCID and its effect is found in evolutionary psychology. Women have more to lose from not thinking carefully in matters of mating than men do and this loss is more costly in the context of commitment. Therefore, women have a more reasoned approach to these matters. When encountered cues (e.g., sexual attractiveness) are at odds with preferred cues (e.g., wealth, nurturing abilities), attitudes become more negative. This result is opposite of what was found in Dahl et al. (2009) research, in which women were observed to be more positive toward sexual content when they had heightened commitment thoughts. One explanation for this discrepancy in results is that the cognitive load manipulation in Dahl et al.'s research reduced the effect of SCID, and therefore its likely consequences. Alternatively, divergent results may be accounted for by the particular cues promoted in Dahl et al.'s ads. Depicting a copulating couple, these ads indicate that the male model is popular among women. Popularity is a cue of wealth and nurturing abilities, which are exactly what women value in the commitment context, hence their favorable attitudes.

Intra-sex differences are observed for women, but not for men. Women with high sociosexuality scores have attitudes that are more negative toward the female model ad in the commitment condition than in the control condition. Women with low scores are either marginally more positive in the commitment condition or show no difference across conditions. A likely explanation is that women with high sociosexuality scores are more sensitive to competitive forces at the mating scene than low-score women are. Hence, an attractive female model will possibly be regarded as a competitive force and potential threat to women with heightened commitment thoughts. Although not directly at odds with previously reported results, which did not involve commitment and sociosexuality simultaneously, the current results run somewhat contrary to previous ones. Sengupta and Dahl (2008) found that a positive general attitude toward sex makes women more positive toward sexual ad content, whereas this research's results suggest that high desire is associated with negative ad attitudes. Again, the fact that participants in Sengupta and Dahl (2008) study made their judgments under high cognitive load may have reduced any effect of SCID on attitudes. Because the previously reported findings were produced by an ad portraying a copulating couple, a cue of the male model's popularity was made salient. Women holding generally favorable attitudes toward sex are more sensitive to what they want from a man, even when they are not in a commitment situation. A popular and hence potentially healthy male model should therefore be favorably evaluated by women.

These results also have some implications for advertisers. Although sexual content that is relevant to the advertised product is believed to justify the use of sex appeals in the eyes of consumers, the results reported here show that this is not always the case. When the context reminds the female audience about commitment, they are more negative toward male sexual content even when it is relevant to the advertised product. For men, attitudes toward ads with female sexual content are unaffected by context. Therefore, advertisers should be cautious when targeting female audiences with sexual ad content.

The results presented in the two experiments are also consistent with a socialization-based explanation for women's and men's differential reactions to sexual content. In particular, it could be that the happiness condition, rather than the parenting thoughts or the romantic arousal condition, drives the result for women. Possibly, sexual images are perceived as more acceptable in happiness contexts because happiness might lessen some of the constraints society imposes on women. Further, women who score high on sociosexuality will more easily be influenced by a happiness prime, because these women are initially less constrained.

## Author contributions

The author confirms being the sole contributor of this work and approved it for publication.

## Funding

Data collection for this research was funded by Department of Marketing, BI Norwegian Business School's research fund.

### Conflict of interest statement

The author declares that the research was conducted in the absence of any commercial or financial relationships that could be construed as a potential conflict of interest.

## References

[B1] Bailey J. M. Gaulin S. Agyei Y. Gladue B. A. (1994). Effects of gender and sexual orientation on evolutionarily relevant aspects of human mating psychology. J. Pers. Soc. Psychol. 66, 1081–1093. 10.1037/0022-3514.66.6.1081 8046578

[B2] Baumeister R. F. (2000). Gender differences in erotic plasticity: the female sex drive as socially flexible and responsive. Psychol. Bull. 126, 347–374. 10.1037/0033-2909.126.3.347 10825779

[B3] Baumeister R. F. Twenge J. M. (2002). Cultural suppression of female sexuality. Rev. Gen. Psychol. 6, 166–203. 10.1037/1089-2680.6.2.166

[B4] Buss D. M. (1996). Paternity uncertainty and the complex repertoire of human mating strategies. Am. Psychol. 51, 161–162. 10.1037/0003-066X.51.2.161

[B5] Buss D. M. (1998). Sexual strategies theory: historical origins and current status. J. Sex Res. 35, 19−31. 10.1080/00224499809551914

[B6] Buss D. M. Schmitt D. P. (1993). Sexual strategies theory: an evolutionary perspective on human mating. Psychol. Rev. 100, 204–232. 10.1037/0033-295X.100.2.204 8483982

[B7] Campbell M. C. Kirmani A. (2000). Consumers' use of persuasion knowledge: the effects of accessibility and cognitive capacity on perceptions of an influence agent. J. Consum. Res. 27, 69−83. 10.1086/314309

[B8] Dahl D. W. Sengupta J. Vohs K. D. (2009). Sex in advertising: gender differences and the role of relationship commitment. J. Consum. Res. 36, 215–231. 10.1086/597158

[B9] Fiske S. Taylor S. E. (1991). Social Cognition. New York, NY: Mc-Graw Hill.

[B10] Fitzsimons G. J. (2008). A death to dichotomizing. J. Consum. Res. 35, 5–8. 10.1086/589561

[B11] Geer J. H. Bellard H. S. (1996). Sexual content induced delays in unprimed lexical decisions: gender and context effects. Arch. Sex. Behav. 25, 379–395. 10.1007/BF02437581 8836471

[B12] Geer J. H. Manguno-Mire G. M. (1996). Gender differences in cognitive processes in sexuality. Annu. Rev. Sex. Res. 7, 90–124.

[B13] Geer J. H. Melton J. S. (1997). Sexual content-induced delay with double-entendre words. Arch. Sex. Behav. 26, 295–316. 10.1023/A:1024574915201 9146815

[B14] Griffitt W. Kaiser D. L. (1978). Affect, sex guilt, gender, and the rewarding-punishing effects of erotic stimuli. J. Pers. Soc. Psychol. 36, 850−858. 10.1037/0022-3514.36.8.850

[B15] Gross J. J. (1998). Antecedent-and response-focused emotion regulation: divergent consequences for experience, expression, and physiology. J. Pers. Soc. Psychol. 74, 224–237. 10.1037/0022-3514.74.1.224 9457784

[B16] Hayes A. F. (2013). Introduction to Mediation, Moderation, and Conditional Process Analysis: A Regression-Based Approach. New York, NY: Guilford Press.

[B17] Hill C. A. (2002). Gender, relationship stage, and sexual behavior: the importance of partner emotional investment within specific situations. J. Sex Res. 39, 228–240. 10.1080/00224490209552145 12476270

[B18] Huff C. Tingley D. (2015). “Who are these people?:” Evaluating the demographic characteristics and political preferences of MTurk survey respondents. Sage Res. Pol. 1, 1–12. 10.1177/2053168015604648

[B19] Kenrick D. T. Groth G. E. Trost M. R. Sadalla E. K. (1993). Integrating evolutionary and social exchange perspectives on relationships: Effects of gender, self-appraisal, and involvement level on mate selection criteria. J. Pers. Soc. Psychol. 64, 951–969. 10.1037/0022-3514.64.6.951

[B20] Kinsey A. C. Pomeroy W. B. Martin C. E. (1948). Sexual Behavior in the Human Male. Philadelphia, PA: Saunders.

[B21] Kinsey A. C. Pomeroy W. B. Martin C. E. Gebhard E. (1953). Sexual Behavior in the Human Female. Philadelphia, PA: Saunders.

[B22] Levay K. E. Freese J. Druckman J. N. (2016). The demographic and political composition of mechanical turk samples. SAGE Open 6, 1–17. 10.1177/2158244016636433

[B23] Lippa R. A. (2009). Sex differences in sex drive, sociosexuality, and height across 53 nations: testing evolutionary and social structural theories. Arch. Sex. Behav. 38, 631–651. 10.1007/s10508-007-9242-8 17975724

[B24] Majoor M. (2011). Gender, Need for Cognition and Product Category Involvement as Influencers of Responses to Advertising. Master's thesis, Oslo, BI Norwegian School Of Management.

[B25] Malamuth N. M. (1996). Sexually explicit media, gender differences, and evolutionary theory. J. Commun. 46, 8−31. 10.1111/j.1460-2466.1996.tb01486.x

[B26] Maner J. K. Gailliot M. T. Rouby D. A. Miller S. L. (2007). Can't take my eyes off you: attentional adhesion to mates and rivals. J. Pers. Soc. Psychol. 93, 389–401. 10.1037/0022-3514.93.3.389 17723055

[B27] Mayer J. D. Gaschke Y. N. (1988). The experience and meta-experience of mood. J. Pers. Soc. Psychol. 55, 102–111. 3418484 10.1037//0022-3514.55.1.102

[B28] Millar M. G. Ostlund N. M. (2006). The effects of a parenting prime on sex differences in mate selection criteria. Pers. Soc. Psychol. Bull. 32, 1459–1468. 10.1177/0146167206291340 17030888

[B29] Penke L. Asendorpf J. B. (2008). Beyond global sociosexual orientations: a more differentiated look at sociosexuality and its effects on courtship and romantic relationships. J. Pers. Soc. Psychol. 95, 1113–1135. 10.1037/0022-3514.95.5.1113 18954197

[B30] Petty R. E. Cacioppo J. T. Heesacker M. (1981). Effects of rhetorical questions on persuasion: a cognitive response analysis. J. Pers. Soc. Psychol. 40, 432–440. 10.1037/0022-3514.40.3.432

[B31] Paolacci G. Chandler J. Ipeirotis P. G. (2010). Running experiments on Amazon Mechanical Turk. Judgm. Decis. Mak. 5, 411–420.

[B32] Rupp H. A. Wallen K. (2008). Sex differences in response to visual sexual stimuli: a review. Arch. Sex. Behav. 37, 206–218. 10.1007/s10508-007-9217-9 17668311 PMC2739403

[B33] Schmitt D. P. (2005). Sociosexuality from Argentina to Zimbabwe: a 48-nation study of sex, culture, and strategies of human mating. Behav. Brain Sci. 28, 247–275. 10.1017/S0140525X05000051 16201459

[B34] Schmitt D. P. (2007). Sexual strategies across sexual orientations: how personality traits and culture relate to sociosexuality among gays, lesbians, bisexuals, and heterosexuals. J. Psychol. Human Sex. 18, 183–214. 10.1300/J056v18n02_06

[B35] Schwartz P. Rutter V. E. (1998). The Gender of Sexuality. Thousand Oaks, CA: Pine Forge Press.

[B36] Sengupta J. Dahl D. W. (2008). Gender-related reactions to gratuitous sex appeals in advertising. J. Consum. Psychol. 18, 62–78. 10.1016/j.jcps.2007.10.010

[B37] Simpson J. A. Gangestad S. W. (1991). Individual differences in sociosexuality: evidence for convergent and discriminant validity. J. Pers. Soc. Psychol. 60, 870–883. 1865325 10.1037//0022-3514.60.6.870

[B38] Solomon M. R. Ashmore R. D. Longo L. C. (1992). The beauty match-up hypothesis: congruence between types of beauty and product images in advertising. J. Advert. 21, 23–34. 10.1080/00913367.1992.10673383

[B39] Spiering M. Everaerd W. Laan E. (2004). Conscious processing of sexual information: mechanisms of appraisal. Arch. Sex. Behav. 33, 369–380. 10.1023/B:ASEB.0000028890.08687.94 15162083

[B40] Till B. D. Busler M. (2000). The match-up hypothesis: physical attractiveness, expertise, and the role of fit on brand attitude, purchase intent and brand beliefs. J. Advert. 29, 1–13. 10.1080/00913367.2000.10673613

[B41] Trivers R. L. (1972). Parental investment and sexual selection, in Sexual Selection and the Descent of Man, ed Campbell B. (Chicago, IL: Biological Laboratories, Harvard University), 136–179.

